# Diminished Anthropometric Measures and Other Associated Variables in a Sample of Violent Offenders: A Case-Control Study

**DOI:** 10.7759/cureus.53475

**Published:** 2024-02-02

**Authors:** Laura J García-Mendoza, Ana Laura Calderón-Garcidueñas, Ruben Ruiz-Ramos, Octavio Carvajal-Zarrabal, Patricia B Denis-Rodríguez, Laura M Bolívar-Duarte, Noé López-Amador

**Affiliations:** 1 Master’s Degree Program in Forensic Medicine (SNP-CONAHCYT), University of Veracruz, Boca del Río, MEX; 2 Department of Neuropathology, National Institute of Neurology and Neurosurgery, Mexico City, MEX; 3 Faculty of Medicine (Veracruz), University of Veracruz, Veracruz, MEX; 4 Institute of Forensic Medicine, University of Veracruz, Boca del Río, MEX; 5 Faculty of Bioanalysis, University of Veracruz, Veracruz, MEX

**Keywords:** case-control study, inmate, d2:d4 finger length ratio, low stature, criminogenic risk factor

## Abstract

Background

Predicting criminal behavior is a complex task due to its multidimensional nature. Nevertheless, health professionals and criminologists must consider individual criminogenic risk factors to provide reliable expert opinions. Physical traits have been a subject of scrutiny since the inception of biological positivism.

Aim

The main objective of this study is to analyze differences in individual characteristics between violent offenders and healthy volunteers to potentially identify predictors of criminal behavior.

Methods

We conducted a case-control study with a sample of inmates convicted of violent offenses and compared them to healthy volunteers. Anthropometrics, sociodemographic data, drug consumption, characteristics of the family nucleus, clinical background, and basic laboratory test results were collected. Quantitative data were tested for normality and homogeneity before applying the Mann-Whitney or T-Student tests, respectively. For categorical data, Pearson’s chi-square test was used for associations, and the odds ratio was determined for the associated risk in drug abuse profiles.

Results

Among the male participants (N = 72), the inmate group (n = 41) showed significantly lower stature (mean height [m]: 1.7454 ± 0.0694 vs 1.6643 ± 0.0659, p < 0.001), a reduced left D2:D4 finger length ratio (mean ratio [cm]: 0.9638 ± 0.0572 vs 0.9380 ± 0.068cm, p < 0.05), and smaller anthropometric measurements, including armful (mean length [m]: 1.8080 ± 0.7690 vs 1.6582 ± 0.7250, p < 0.001), wrist (mean [cm]: 17.39 ± 1.10 vs 16.57 ± 1.84, p < 0.05), mid-upper arm (mean [cm]: 31.75 ± 3.79 vs 29.97 ± 3.79, p < 0.05), and head circumferences (mean [cm]: 58.43 ± 1.92 vs 55.39 ± 1.51, p < 0.001). Additionally, the inmate group exhibited shorter lower segments (mean [cm]: 102.67 ± 4.97 vs. 97.85 ± 5.04, p < 0.001) and plantar lengths (mean [cm]: 27.45 ± 1.25 vs. 26.78 ± 1.00, p < 0.05). Furthermore, this group displayed a higher risk of alcohol (OR = 4.4, p < 0.01), cocaine (OR = 3.36, p < 0.05), and benzodiazepine consumption (OR = 3.36, p < 0.05). Parental alcohol consumption (χ² = 12.66, p < 0.01) and the practice of Protestantism (χ² = 20.087, p < 0.001) were also associated with the inmate group.

Conclusion

Physical traits may be considered potential criminogenic risk factors, but larger studies are necessary to validate these findings. Future research should take into account physiological and psychological correlates to gain a comprehensive understanding of the complex relationship between physical traits and criminal behavior.

## Introduction

The prevention of violent crime through early diagnosis of antisocial and criminal behavior is considered one of the most significant contributions of criminology and behavioral science. Achieving an accurate diagnosis requires a multidimensional approach for each case. In this regard, biological positivism sought to use physical and biological traits to predict criminogenic risk [1]. During the late 19th century, Cesare Lombroso's work heavily influenced classical biological positivism, also known as criminal anthropology. Lombroso's theories centered around the concept of atavism as a cause of criminal behavior. He proposed specific physical and mental traits as predictors for violent criminals, referring to this phenotype as the 'criminal man' [2]. However, his theories faced strong opposition from other criminologists who argued that concepts like atavism and Lombroso's hypotheses were discriminatory and lacked scientific basis, aligning with Beccaria's vision of crime [1,3]. Despite these controversies, this criminological standpoint has persisted throughout time.

In the latter half of the 20th century, as molecular genetics and neuroscience advanced, a form of neolombrosianism gradually gained acceptance among some scholars, acknowledging the involvement of certain genetic traits in criminal behavior [4]. Jacobs' syndrome, a sexual chromosome trisomy (47-XYY), is characterized by a phenotype that includes macroorchideae, tall stature, macrocephaly, and hypertelorism. Individuals with this syndrome have a higher susceptibility to conditions such as asthma, autism spectrum disorder, and seizures [5,6]. This syndrome sparked discussions concerning a new criminogenic trait theory, evoking memories of Lombroso's atavism, and even led to debates on bioethics and genetic counseling, with some physicians proposing eugenic perspectives [7].

Furthermore, Brunner et al. [8] reported on a cohort of five male subjects from a Dutch family exhibiting common traits of borderline intellectual disability and abnormal behavior, including pyromania, aggression, sexual violence, and exhibitionism. These individuals displayed excessive concentrations of monoamines in their urine, attributed to deficient activity of the enzyme monoamine oxidase-A (MAO-A), responsible for biogenic amine metabolism. The researchers identified a point mutation in exon 8 of the MAO-A structural gene, leading to the substitution of glutamine for a termination codon, thus giving rise to the eponymous syndrome. This genetic and metabolic abnormality sheds light on the underlying factors contributing to the observed behavioral manifestations.

Other studies have contributed to the expanding list of genetic abnormalities associated with aberrant or criminal behavior. For instance, the Val158Met mutation of the catechol-O-methyltransferase (COMT) gene has been linked to various disorders, including schizophrenia [9], bipolar disorder [10], and alcoholism [11]. These findings, in conjunction with other research [12], underscore the significant role of genetic factors in influencing such behaviors.

A search performed in the Gene Bank database of the National Center for Biotechnology Information at the National Library of Medicine in the USA unveiled a minimum of 19 human genes linked to mutations associated with criminal, antisocial, and violent behavior. It is worth highlighting that a number of these genes have direct or indirect connections to the metabolism of sex steroids, specifically androgens (MAOA, AR, DRD1, DAT, DRD3, and DRD4) [13].

The evidence presented so far lays the foundation for further exploration of physical characteristics, their genotypes, and physiological correlations, offering a fresh biological perspective on criminal behavior. This study aims to investigate physical traits associated with violent behavior and asks whether these traits can predict criminal propensity.

Through the analysis of physical characteristics and their genetic and physiological components, researchers may discover biomarkers or cues linked to a higher risk of criminal behavior. This approach recognizes the influence of biology on human behavior and aims to improve our understanding of the complex interplay between genetics, physiology, and criminal tendencies.

In pursuit of our objective, we compared a group of convicted violent offenders with individuals lacking criminal records. We focused on identifying differences in anthropometrics, sociodemographic data, clinical histories, and laboratory test results. Our goal was to uncover criminogenic risk indicators. It is important to note that this study followed Strengthening the Reporting of Observational Studies in Epidemiology (STROBE) guidelines for transparency and methodological precision in our approach and reporting.

## Materials and methods

Study design

Prior to the start of the study, approval was obtained from the Ethics in Research Committee of the Institute of Forensic Medicine of the University of Veracruz (CEI2013-03). The procedures used in this study adhere to the tenets of the Declaration of Helsinki. We conducted a descriptive case-control study to compare physical, clinical background, and basic laboratory differences between two groups: convicts who were firmly sentenced for serious violent felonies and individuals without criminal records, social misconduct, or mental illness.

Settings

Volunteers were recruited from two settings: state penitentiary inmates and the community. The Subdirectorate of Social Rehabilitation of the State of Veracruz, Mexico, facilitated the study in two different prisons. Inmates were informed about the study, attended a presentation, and provided an informed consent form. Community volunteers were invited through direct contact, social media, posters, and flyers. Interested individuals contacted the team for an interview and signed the consent form. Sample collection took place at the laboratory, followed by a clinical instrument assessment for data gathering and a physical examination.

Participants

Male adult volunteers convicted for serious violent felonies (e.g., homicide, rape, kidnapping) were recruited for the case group. Male adult volunteers from the community without criminal records, social misconduct, or severe mental illness were recruited for the control group. All volunteers were adults over the age of 18.

Variables

A clinical instrument with 168 predictors collected sociodemographic data (e.g., age, birthplace, education), family characteristics, medical history, physical examination, drug abuse, and criminological background. Basic laboratory tests (e.g., blood count and glucose levels) were performed. Variables were categorized as categorical, ordinal, or continuous for analysis using appropriate statistical tests.

Data sources

Sociodemographic data and clinical background information were obtained through individual interviews using a clinical instrument. A physical examination measures various parameters such as blood pressure, pulse, temperature, respiratory rate, stature, weight, body mass index, head circumference, waist circumference, and more. The D2:D4 finger length ratios were calculated. Blood samples were collected from individuals in a fasting state through a venipuncture in the cephalic vein. The samples were sent to the laboratory for analysis, including blood count, chemistry, lipid profile, renal and hepatic function, and blood type determination. All data were compiled into a CSV file for analysis using R Studio.

Bias

This study was conducted directly by the multidisciplinary team with the participation of volunteers, ensuring data obtained from primary sources and minimizing biases associated with relying on secondary or tertiary sources. However, as with any study, there is a possibility of data capture or interpretation errors in certain cases. Efforts were made to maintain objectivity and transparency, but variables relying on volunteers' honesty and cooperation may introduce bias.

A special type of selection bias could be present in this study due to the dependence on judicial sentences for the expected outcome, making it difficult to verify procedural errors in the administration of justice. This bias is expected to be similar throughout the state where the study was conducted but may vary across regions and countries. Any other identified biases in the study results will be thoroughly discussed in the corresponding section.

Study size

The number of volunteers recruited from authorized penitentiaries and the open community was based on their willingness to participate. Efforts were made to maintain group homogeneity, but variables like age and educational level resulted in some unavoidable differences between the groups.

The sample for this study was drawn from a population of 2,213,427 individuals residing in the metropolitan areas of Xalapa, Veracruz, Orizaba, and Córdoba, which fall under the jurisdiction of the penitentiaries "Amatlán" and "Pacho Viejo." Given the large population size, a margin of error of 11.32%, a 95% confidence level, and a presumed population proportion of 40% for males, a sample size of N = 72 observations was deemed sufficient to obtain a representative sample for the region.

Quantitative variables

All quantitative variables were treated as raw numbers to determine the mean and standard deviation values.

Statistical methods

Continuous variables

To analyze continuous variables, we assessed normality using the Kolmogorov-Smirnov test and checked for homogeneity of variances using the Levene test. For mean comparisons between groups, we calculated standard deviations. If the data did not follow a normal distribution or if variances were not homogeneous, we used the Mann-Whitney test. If the data followed a normal distribution and variances were homogeneous, we employed the t-test (for independent samples). These statistical methods were used to identify significant differences in continuous variables between the investigated groups.

Categorical data

To assess the risk of drug abuse, we calculated odds ratios for each substance in relation to the study groups and determined the frequencies of drug abuse within each group. For categorical data, such as religious belief or medical background, we used Pearson's chi-square test to examine associations between variables and determine significant associations between categories. These methods allowed us to investigate potential risk factors, associations related to drug abuse, and relationships between categorical variables in our study.

Data processing

Data from interviews were coded and transferred to a CSV database file with 168 observations and 72 registers. Statistical analysis was conducted using RStudio Desktop IDE software (version 2023.06.0 + 421) on MacOS11+ operating systems. A 95% confidence interval was used, and statistical significance was determined with a p-value threshold of 0.05 (p < 0.05).

## Results

Participants

After confirming eligibility, a total of 72 (N, 100%) volunteers were included in this study. Among them, 41 (56.9%) individuals had been convicted for serious felonies and violent crimes in the case group, specifically qualified homicide (n=12, 16.6%), rape (n=9, 12.5%), kidnapping (n=4, 5.5%), aggravated assault (n=11, 15.2%), qualified robbery (n=3, 4.1%), and violent abuse of authority (n=2, 2.7%). The determination of their convictions was based on definitive court resolutions. In the control group, 31 (43.1%) individuals were included from the community with no criminal records, serious offenses, or mental illness in their personal backgrounds. 

Confidentiality of the provided data was ensured through the informed consent form, which clearly outlined the objectives of the study and emphasized the voluntary nature of participation. These measures were implemented in accordance with the recommendations of the Ethics in Research Committee of the Institute of Forensic Medicine. All 72 volunteers willingly signed the consent form prior to sharing their data and providing a blood sample.

Descriptive data

All participants were adult males aged 18 years and older, with no upper age limit. There were significant differences in mean age between the groups (inmates: 39.26 ± 11.16, controls: 28.61 ± 10.37, W = 241, p = 0.000007) due to uncontrolled participation. However, this age difference did not impact bone development, as most participants had already completed their bone development process. It is important to note that younger controls may still have some growth remaining, resulting in taller heights. The difference in age did affect variables like educational level, as younger adults in the community have access to continuous education and other benefits. Hence, variables influenced by age differences were not considered in the analysis. Unfortunately, laboratory results for 12 participants (including one inmate) were either lost or the controls did not show up for sample collection.

Main results

Our clinical instrument for recording data consists of 168 observations (variables and predictors) that were meticulously coded prior to performing the statistical analysis. To facilitate the reporting of results, we only present data that have sufficient statistical power (95% confidence interval) and significance (p < 0.05). Therefore, mean differences and associations that are not statistically significant will not be shown. Each group of observations will be reported in separate sections. Variables that did not achieve statistical significance but might be relevant for interpreting the study results, or those that were excluded due to having a direct or indirect relationship with age, are enumerated in Table 3 as supplementary material.

Inmates Exhibited Significantly Smaller Anthropometric Measurements Compared to the Control Group

Surprisingly, as shown in Table 1, there were significant differences in anthropometric measurements between the groups. Inmates had a lower stature, left D2:D4 finger length ratio, smaller arm, wrist, mid-upper arm, head circumferences, lower segment, and plantar lengths compared to the control group. It is worth noting that the ethnic composition of the population in the study area showed no statistically significant differences and was particularly homogeneous due to the prevalent mestizaje (ethnic mixture) in this region of Mexico. In other words, there is no dominance of a specific ethnicity, and certainly no group with greater 'racial purity'.

**Table 1:** Significant anthropometric differences between groups Values are given in mean ± standard deviation (SD). ***p < 0.001, *p < 0.05, ns = not significant.

Anthropometrics	Mean (X) ± SD	
	Control (*n* = 31)	Inmate (*n* = 41)
Stature (m)	1.7454 ± 0.0694	1.6643 ± 0.0659***
Right D2:D4 ratio (cm)	0.9620 ± 0.0604	0.9401 ± 0.0612 ns
Left D2:D4 ratio (cm)	0.9638 ± 0.0572	0.9380 ± 0.068*
Armful (cm)	180.80 ± 7.69	165.82 ± 7.25 ***
Wrist (cm)	17.39 ± 1.10	16.57 ± 1.84*
Mid-upper arm (cm)	31.75 ± 3.79	29.97 ± 3.79*
Head (cm)	58.43 ± 1.92	55.39 ± 1.51***
Lower segment (cm)	102.67 ± 4.97	97.85 ± 5.04***
Plantar length (cm)	27.45 ± 1.25	26.78 ± 1.00*

Inmates Had a Higher Risk of Alcohol, Cocaine, and Benzodiazepine Consumption Compared to Controls

In the inmate group, there was a 4.4-fold increased risk of alcohol and a 3.36-fold increased risk of cocaine and benzodiazepine consumption, as compared to controls. However, the consumption of other substances such as tobacco, cannabis, opioids, amphetamines, lysergic acid, or solvents did not reach statistical significance in this study.

Interestingly, the inmate group exhibited a significantly higher risk of previous drug rehabilitation prior to imprisonment, while none of the controls had a history of drug rehabilitation at the time of inclusion in this study (Table 2).

**Table 2:** Risks of substance consumption and rehabilitation associated to violent criminal behavior OR: odds ratio, CI: confidence interval, inf: Infinite, ns: not significant, *p < 0.05, **p < 0.01, ***p < 0.001.

	OR	CI (95%)	p -value
Alcohol	4.40	1.53–13.83	0.005**
Tobacco	2.23	0.84–6.09	0.10 ns
Cannabis	0.88	0.33–2.33	0.80 ns
Cocaine	3.36	1.03–13.53	0.04 *
Benzodiazepines	3.36	1.03–13.53	0.04*
Opioids	0.75	0.01–30.18	0.86 ns
Amphetamines	1.44	0.11–46.80	0.78 ns
LSD	inf	0.51– inf	0.09 ns
Solvents	1.86	1.49–2.32	0.053 ns
Previous rehab	inf	5.09–inf	0.000004***

Religion, Parental Addiction, and Other Variables

**Figure 1:**
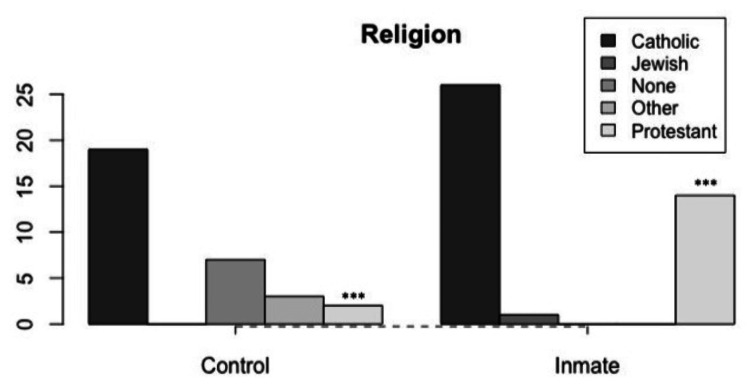
Differences in religious beliefs The data has been represented as frequency (N=72). The inmate group showed a higher association with the Protestant religion (X^2^ = 20.087, df = 4, ***p < 0.001).

**Figure 2:**
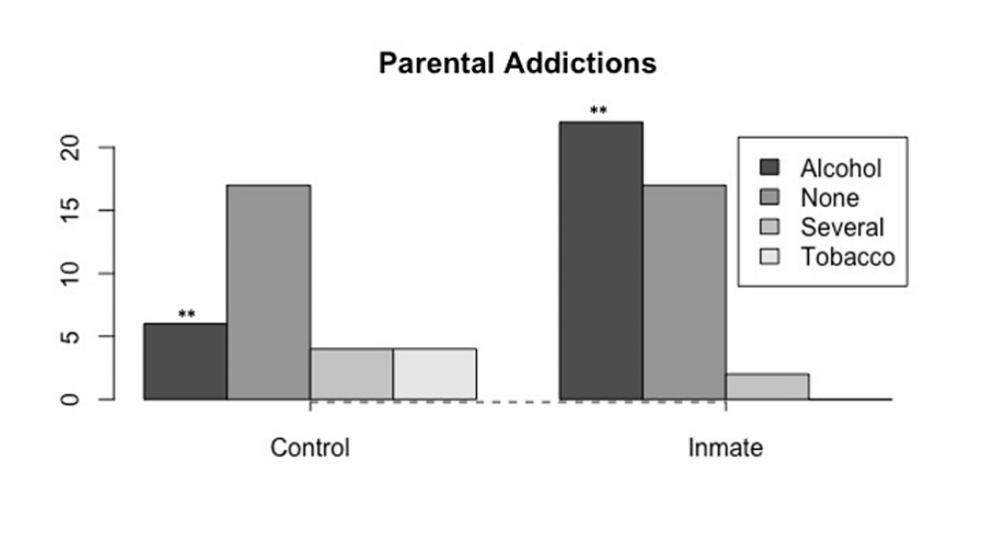
Parental substance abuse profiles The data have been represented as frequency (N=72). Parental alcohol consumption was strongly associated with the inmate group (X^2^ = 12.66, df = 3, **p < 0.01).

An analysis of categorical data revealed a clear association between the inmate group and the practice of Protestantism (Figure 1), as well as parental alcohol consumption (Figure 2). Despite the inmate group being older in age than the controls, no significant differences were found in terms of clinical background, physical examination, or laboratory tests. However, it is worth mentioning that inmates had lower diastolic blood pressure (inmate: 70.82 ± 12.33, control: 81.70 ± 7.67, W = 1039.5, p = 0.000003) and fasting glucose levels (inmate: 81.21 ± 11.62, control: 95.46 ± 7.63, t = 6.05, df = 64.88, p = 0.00000007) compared to controls. Both results could be considered expected, given the caloric and sodium restrictions in the diet of the inmate population.

## Discussion

The fundamental objective of this study was to juxtapose a variety of predictors sourced from diverse information channels between individuals convicted of severe violent crimes and those without criminal records. These predictors encompassed an array of dimensions, including sociodemographic data, anthropometric measurements, family nucleus attributes, clinical histories, physical examinations, and rudimentary laboratory tests. The identification of noteworthy differences and associations within these variables could potentially posit them as prospective predictors of criminal behavior. However, it is imperative to note that further substantiation through more extensive studies is imperative. The all-encompassing scope of this study was meticulously structured to address the overarching query: Can physical traits be harnessed for the assessment of criminal behavior risk? The ensuing sections are dedicated to illuminating the pivotal findings of this study and their pertinence to the central research inquiry.

Physical characteristics as criminogenic risk factors: neolombrosianism or discrimination?

Considering the observed differences in anthropometric measurements, there may be a temptation to regard these variations as potential predictors. Nevertheless, it is imperative to acknowledge that establishing such a correlation is a complex undertaking. Notably, Lombroso's 'criminal man' theory, positing that criminals were taller and heavier than their noncriminal counterparts [2], stands in contrast to our findings. Our study reveals that inmates convicted of violent crimes exhibit a smaller osteomuscular constitution, thereby contradicting Lombroso's assertions. It is essential to recognize that Lombroso's research has encountered criticism for its lack of scientific rigor from scholars both in historical and contemporary contexts [14,15]. Consequently, prudence should prevail when making direct comparisons or formulating assumptions based on these historical theories.

It is of paramount importance to underline that our findings must not be extrapolated to suggest any inherent criminal predisposition or to attach negative implications to individuals of smaller physical stature. Prins and Reich [16], in a systematic review with a meta-analysis, underscored the inconsistent nature of statistics used in research related to criminogenic risk assessment. As a result, any new criminogenic risk factor or predictive marker proposed must satisfy stringent criteria to be deemed valid. Instead, our study illuminates an intriguing pattern that beckons for deeper investigation and exploration.

It is conceivable that certain traits observed in violent offenders could stem from changes occurring during the prepubertal period. Premature epiphyseal closure during bone development might arise due to disruptions in sexual steroid metabolism in this phase, as seen in cases of steroid abuse and certain conditions [17-19]. An imbalance in sexual steroid metabolism could potentially influence neurodevelopment in late adolescence, leading to anomalous plasticity phenomena that contribute to behaviors like addiction and aggression [20]. Furthermore, Zelleroth [21] conducted an in vitro assay demonstrating that androgens hinder neurite outgrowth and neuronal viability, thus affecting neuronal connectivity.

Within our research team, ongoing discussions have taken place regarding the potential impact of infant malnutrition or pubertal drug consumption on bone and muscle development. However, it is crucial to emphasize that these hypotheses require rigorous testing through suitable experimental methodologies specifically conducted in the adolescent population.

To conduct a thorough assessment of this discovery, it becomes imperative to replicate this study across various regions and countries, considering the adjusted means and percentiles for the height of distinct populations. The availability of anthropometric data facilitates regular scrutiny when new inmates are admitted into jails.

The D2:D4 finger length ratio has been associated with heightened androgenic stimulation during pregnancy. Existing research indicates that androgen levels during pregnancy, influenced by factors such as maternal smoking, exhibit an inverse correlation with this ratio. This observation has given rise to the notion that the D2:D4 finger length ratio could potentially function as an indicator of prenatal androgen exposure, shedding light on hormonal influences during early development that are linked to a broad range of mental conditions. Interestingly, in line with these discoveries, alterations in both early and late development appear to be impacted by sexual steroids [22-24].

The neural basis of addiction and violent crime: sexual steroids, the limbic and reward systems, and parental influences

The limbic system constitutes a sophisticated network of brain structures primarily tasked with the processing of emotions, motivation, and memory. This intricate system encompasses various interconnected regions, among them the amygdala, hippocampus, hypothalamus, and cerebral cortex. Together, these components collaborate to regulate and modulate an array of cognitive and emotional functions. Moreover, the limbic system maintains a fundamental interconnection with the reward system, commonly referred to as the mesolimbic pathway. Within the realm of the reward system, pivotal regions such as the ventral tegmental area, nucleus accumbens, and prefrontal cortex play a pivotal role in orchestrating experiences of pleasure and reinforcement [25].

Sexual steroids, encompassing androgens and estrogens, have been revealed to exert an impact on the operation of both the limbic system and the reward system [26,27]. These hormones possess the capacity to mold the plasticity response within these systems, heightening their interconnections and optimizing various cognitive and behavioral functions. As previously noted, disturbances in the metabolism of sexual steroids can lead to atypical plasticity phenomena, which in turn hold the potential to influence responses related to reward and emotions. These modifications have been implicated in associations with addiction and deviant behavior [28].

Research has established that parental influences play a pivotal role in shaping the interconnected reward and emotional responses governed by the limbic system and the reward system [29,30]. In the context of our study, a compelling correlation emerged between a parental background of alcohol consumption and individuals with a history of serious criminal convictions. The question of whether this potential predictor impacts the evolution of criminal or violent tendencies via conditioning and learning paradigms, or if it signifies an inherited trait intertwined with genetic and epigenetic considerations, remains to be fully understood.

The introduction underscored the presence of a group of 19 genes documented in the Gene Bank, linked to mutations associated with criminal and violent conduct. Within this set of genotypes, specific variations are believed to play a role in modifying the metabolism of biogenic amines, the operation of the hypothalamic-pituitary-gonadal axis, and the regulation of reward and emotional responses. These genetic elements have been identified as potential factors contributing to the emergence of criminal inclinations and aggressive actions [31]. Grasping the repercussions of these genetic variances on neural pathways and neurotransmitter systems is imperative for a comprehensive grasp of the inherent biological mechanisms associated with criminal behavior.

Practicing a religion and crime: is there a connection?

Certain meta-analyses propose that religious convictions and practices exert a discouraging influence on violent and criminal conduct [32,33]. Nevertheless, a unanimous consensus regarding the precise impact of religious beliefs and practices on the occurrence of criminal behavior has yet to be established [34]. Notably, in our investigation, the elevated frequency of Protestantism practice within our convict sample draws attention. However, it is crucial to clarify that within our study, this phenomenon is interpreted as an environmental outcome rather than a predictor.

During discussions with the inmate population, our multidisciplinary team noted a palpable religious milieu within prisons. This circumstance can be attributed to the formation of clusters of religious adherents with Protestant Christian beliefs among the inmates, who have collaborated with penitentiary authorities to aid in the process of social reintegration. Consequently, it is posited that this heightened proportion of Protestant practitioners stems from conversions occurring within inmate groups in prisons. It is important to acknowledge, however, that our questionnaire's design did not anticipate this discovery, potentially introducing bias that necessitates addressing in future research on this subject.

In the end, the connection between religion and criminal activity remains intricate and presents expanding research prospects for delving into the impact of religious phenomena on criminal tendencies.

Limitations of the study

Our study has several limitations that should be noted for a proper contextual interpretation of the data. First, restricted access to correctional facilities due to security concerns limited our interaction with convicts to pre-approved scenarios. Second, our sample size relied on voluntary participation, and while we aimed to reach regional significance, establishing collaboration networks on national and international scales is essential to replicate the study in a broader and more globally representative cohort. Financial constraints, particularly in our country, hindered acquiring more comprehensive data, such as diverse polymorphism prevalence, genetic mutations, and hormone and amine levels within the target population.

Additionally, the lack of psychological investigations to confirm the presence of behavioral disorders is a noteworthy limitation. This was due to restricted prison access, preventing us from conducting a thorough psychological assessment within the allotted timeframe. In future phases, we plan to initiate longitudinal cohort studies to track well-characterized cases and evaluate outcomes over time, including various interventions used. Despite these limitations, it is important to recognize that this study is exploratory, focusing on readily identifiable traits and information gathered through interviews. However, these attributes also represent strengths, as the research's reproducibility is feasible for researchers worldwide.

We encourage other research teams to collaborate and expand the sample size swiftly, improving the generalizability and robustness of the findings. This study provides valuable and intriguing insights into understanding violent and criminal behavior.

Interpretation and generalisability

Until we gain more insights into the biological and behavioral aspects of these findings, the conclusions from this study should be viewed as speculative hypotheses based on empirical data and guided by theoretical implications. Currently, there is no direct research establishing a conclusive link between reduced stature, smaller anthropometric measurements, and a propensity for violent or criminal behavior. It is important to clarify that this study does not claim authoritative outcomes but presents an intriguing proposition and logically deduced hypothesis rooted in a theoretical framework. This hypothesis focuses on the potential impact of hormones during early and later developmental phases, emphasizing the need for further comprehensive investigations.

Detecting abnormal developmental trajectories in the early stages, before violent or criminal tendencies manifest, offers the potential for early prevention and education for at-risk individuals. This responsibility is shared by governments and educational institutions worldwide. The study's strength lies in its wide applicability, as it does not require specialized equipment or expertise. Through broader validation and a global perspective, these discoveries can contribute significantly to criminology and behavioral science.

## Conclusions

Addressing our research question is complex, even after analyzing our empirical data meticulously. Labeling biological traits as potential criminogenic risk factors requires a comprehensive understanding of both the physical attributes and the intricate biological and behavioral associations linked to these phenotypes. The validity of empirical deductions in this area depends on clear trait definitions and a deep understanding of their biological foundations. It is essential to recognize that the environment significantly influences how genotypic traits adapt and manifest. The interplay between genetics and the environment has gained scholarly attention, with a focus on epigenetic investigations.

We are cautious about embracing biological and physical attributes as direct predictors of criminal behavior, as initially proposed by Lombroso. However, deeper exploration of the intricate biological, behavioral, and sociocultural adaptive mechanisms may lead us to a new era in criminology. In this context, biological positivism could potentially contribute to unbiased crime prevention. An objective and scientific approach is crucial, free from bias or preconceived notions. This is essential for a comprehensive understanding of human behavior and its intricate relationship with biological factors.

## Appendices

Supplemental material

**Table 3:** Variables relevant to the study that did not achieve statistical significance or were excluded due to their association with the age of the participants The presented variables encompass various multinomial subcategories that have been scrutinized to identify associations and significant differences between the two study groups. In the first column, we showcase variable groups that did not achieve statistical significance (p>0.05). In the second column, we highlight variable groups that were excluded due to their direct or indirect correlation with the age of the subjects at the time of data acquisition. The database is accessible to interested parties upon a reasonable request directed to the corresponding author.

Variables deemed relevant with p > 0.05	Variables excluded based on age
Language	Romantic partners
Parent’s education	Education
Parent’s occupation	Marital status
Psychiatric disorders in parents	Number of children
Mother's substance abuse	Occupation
Medical history	Family relationship (spouse, children)
Skin color, eyes, hair	
Forehead type, eyebrows, earlobes, nose, lips, chin, facial hair	
Physical build	
Racial dominance	
Language	
Body mass index	
Complete blood count, blood chemistry, and blood type	
